# Vaccine Hesitancy and Refusal Among Parents of Children Aged 5–11 Years: Evidence from the COVID-19 Pandemic in the Calabria Region

**DOI:** 10.3390/vaccines14010017

**Published:** 2025-12-23

**Authors:** Francesca Licata, Concetta Arianna Scicchitano, Emma Antonia Citrino, Aida Bianco

**Affiliations:** 1 Department of Health Sciences, School of Medicine, University of Catanzaro “Magna Græcia”, Viale Europa, 88100 Catanzaro, Italy; f.licata@unicz.it; 2 Department of Medical and Surgical Sciences, School of Medicine, University of Catanzaro “Magna Græcia”, Viale Europa, 88100 Catanzaro, Italy; a.scicchitano@unicz.it (C.A.S.); e.citrino@unicz.it (E.A.C.)

**Keywords:** children, COVID-19 vaccination, Italy, parents, vaccine hesitancy, vaccine refusal

## Abstract

**Background/Objectives:** This study aims to evaluate COVID-19 parental vaccine hesitancy (CPVH) and refusal among parents of children between 5 and 11 years and to identify potential factors influencing them. A secondary aim was to assess knowledge, concerns, and beliefs associated with COVID-19 and immunization. **Methods**: This cross-sectional study was conducted among parents of children between 5 and 11 years using an anonymous, self-administered questionnaire. Sociodemographic characteristics, knowledge, concerns, and beliefs regarding COVID-19 and immunization in children; CPVH according to Parent Attitudes about Childhood Vaccines short scale; COVID-19 vaccination status and intention; and sources of information about COVID-19 vaccination were investigated. **Results**: Among 506 participating parents, only 12.7% correctly answered all six knowledge items. High CPVH was found in 60.1% of respondents and was more prevalent among younger parents and those with lower knowledge levels. Compared to having received no information on COVID-19 vaccination, high CPVH was positively associated with having received information from informal sources and trusting them and negatively associated with information from formal ones. More than half (58.3%) had vaccinated their child, and 38.5% had no intention to vaccinate their child against COVID-19. High CPVH, lower knowledge levels, and a need for further information were significant predictors of vaccine refusal. Conversely, refusal was negatively associated with parental COVID-19 vaccination status, and with having received information from formal and from both formal and informal sources compared to not having received information. **Conclusions:** The findings highlight the need for establishing and investing in platforms to promote vaccine awareness and dispelling misinformation among parents.

## 1. Introduction

The COVID-19 pandemic has had a significant impact on global health systems, economies, and daily life for individuals around the world. In this scenario, mass vaccination campaigns have become a critical component in controlling the spread of the virus and preventing future outbreaks [1]. However, research has indicated that, even in nations with the most developed national health systems, a great percentage of people are hesitant to get vaccinated [2,3]. Vaccine hesitancy (VH) is defined as a “delay in accepting or denying vaccines despite the availability of vaccine services” [4]. In the 2022 position paper published by World Health Organization (WHO) on the behavioral and social drivers of vaccine uptake [5], a new definition of VH “as a motivational state of being conflicted about, or opposed to, getting vaccinated” that precedes and is separated from the actual behavior was proposed. Indeed, VH represents a serious barrier to achieving widespread community immunity [6] and is a long-standing issue that became alarming at the height of the COVID-19 pandemic, fueled by misinformation and amplified through social media channels. COVID-19 VH is a complex issue that stems from several factors, such as environmental (e.g., access barriers), personal (e.g., perception of risk), social (e.g., media and information sources), and safety and vaccine-related factors (e.g., concern about the vaccine development process) [7].

Children infected with COVID-19 usually do not show symptoms at all, remaining undiagnosed, or may show only mild symptoms; nonetheless, some children can become seriously ill with COVID-19. Although it appears children may be at lower risk than adults of developing post-COVID-19 conditions, some do experience serious outcomes, such as long-term COVID and multisystemic inflammatory syndrome in children (MIS-C) [8]. Hence, health authorities also recommended the COVID-19 vaccine in early childhood [9,10]. The efficacy of the mRNA COVID-19 vaccines in children aged 5–11 has been proven. The vaccine was associated with a lower risk of SARS-CoV-2 infections (both symptomatic and asymptomatic), hospitalizations due to COVID–19–related illnesses, and MIS-C [11]. Moreover, the vaccine has been proven to be safe, with no increased risk of serious adverse events [11,12,13].

In December 2021, the Italian Medicines Agency approved the COVID-19 vaccination to include children aged 5 to 11 years old in the vaccination campaign that had already started for adults [14]. However, parents may choose to get vaccinated themselves but may choose not to vaccinate their children, and parental VH (PVH) could represent a significant barrier to achieving stable community immunity [15]. Understanding the reasons for PVH is critical in developing appropriate interventions to increase the COVID-19 vaccine uptake during childhood. Reasons for vaccination of children have been demonstrated to vary depending on region, culture, sex, and other sociodemographic factors [16]. Regarding the COVID-19 vaccine, factors contributing to PVH include a limited understanding of how vaccines work [17], vulnerability to vaccine misinformation [17,18], and underestimation of the severe health risks COVID-19 poses to children [19]. Additionally, some parents believe vaccination is unnecessary, assuming their child is either not susceptible to infection due to good health or unlikely to experience severe illness if infected [17].

Given the significance of vaccination for future pandemic preparedness and response, the first study’s objective was to assess the hesitancy toward and refusal of vaccination against COVID-19 among parents of children between 5 and 11 years; secondary objectives were to describe the sociodemographic characteristics of the sample included in the study and to evaluate the knowledge, concerns, and beliefs associated with COVID-19 and immunization as possible determinants of PVH and refusal.

## 2. Materials and Methods

### 2.1. Study Design and Setting

This cross-sectional study was conducted between May and September 2022 among a sample of parents in Calabria, a region of Southern Italy. The study reporting complies with the Strengthening the Reporting of Observational Studies in Epidemiology (STROBE) standards for reporting cross-sectional studies [20].

### 2.2. Study Population and Data Collection

To be eligible, parents had to be a minimum of 18 years old and have at least one child between the ages of 5 and 11 years. Parents not able to understand, speak, and read Italian were excluded from the enrollment.

A cluster sampling strategy was employed to recruit the participants, with schools as cluster units. First, schools in which 5–11-year-old students may be enrolled were sampled. In Italy, primary education is compulsory from the age of 6, but children aged 5 who turn 6 by 30th April of the same school year can request early entrance. Therefore, we choose to approach and invite parents of those children enrolled in primary schools. To select the cluster units, the region was divided into three main geographical areas (i.e., north, center, and south) and, from each area, 2 schools were randomly selected from a publicly available frame [21] of public and private primary schools (no. 225) in the region. Replacement schools for each sampled school were simultaneously identified in case an originally sampled school chose not to participate (response rate of 50%). In each selected school, the principal was contacted to present the project and to obtain permission and collaboration to conduct the study. A notice was posted on the school’s website home page and electronic register of classes enrolling children between 5 and 11 years, describing the study’s objectives and importance and the procedures to participate and providing a link to complete the questionnaire. In addition, to reach participants who did not use the Internet and to increase the coverage of the target population, data collection was also carried out using a paper questionnaire, provided during 3 sports and cultural events involving children of the target age range within the selected schools. Written consent was obtained from all the parents recruited at those events.

The study’s objectives and procedures were clarified to the parents on the first page of the online and paper questionnaires. It was specified that for each child, only one parent may complete the questionnaire. In the case of the parent having multiple children within the selected age range, it was indicated that they should complete the questionnaire with their youngest child in mind. It was assured that all data would be kept confidential and assessed anonymously and that participation in the study would be entirely voluntary. Furthermore, parents had the discretion to reject or interrupt their participation at any time and without explanation. The participants received neither gifts nor incentives for participation. The questionnaires where more than 20% data were missing were excluded from the study.

### 2.3. Survey Instrument and Outcomes

Building upon previous research on vaccination knowledge and VH [15,22,23,24,25,26,27,28], the research team designed a questionnaire that consisted of five sections and a total of 33 items and took about 15 min to complete. The first section gathered sociodemographic information regarding the parent (i.e., sex, age, marital status, number of children, level of education, and employment status with a specific focus on employment in healthcare sector) and their children (i.e., sex, age). The second section aimed to assess parents’ knowledge regarding COVID-19 and vaccination in children (6 items in a “true”, “false”, or “I do not know” response format). A knowledge score was computed by assigning one point for each right response and summing the scores for each statement (range 0–6). In the third section, parents expressed their concerns and beliefs regarding the risk of COVID-19 and immunization in children (1 item on a ten-point Likert scale, ranging from 1 “not worried” to 10 “very worried”; 2 items on a five-point Likert scale, from “strongly agree” to “strongly disagree”; and 1 item in a “yes” or “no” response format). Furthermore, the Parent Attitudes about Childhood Vaccines short scale (PACV-5) [29] adapted for COVID-19 vaccination [27] was included in this section to measure COVID-19 PVH (CPVH) across three domains: vaccination behavior, general attitude, and trust and beliefs regarding vaccine safety and efficacy (5 items on a five-point Likert scale). The total score ranged from 0 to 10, and parents were classified as low-hesitancy (0–4), moderately hesitant (5–6), or highly hesitant (7–10) on COVID-19 vaccination. The fourth section (7 closed-ended questions with multiple answers) explored the COVID-19 vaccination history of the parent and their child and the intentions, if any, to vaccinate them, along with reasons for their decision. When parents declared that they had not vaccinated their child or had the intention to do so, they were defined as COVID-19-vaccination-refusing parents. Lastly, the final section collected data on vaccination information sources and whether parents felt in need of additional information. The considered sources of information included informal sources (i.e., mass media, social media, the Internet, family, and friends), and formal sources [i.e., the pediatrician, other healthcare providers (HCPs), and national (e.g., the Italian Ministry of Health and the National Health Institute) and international health organizations (e.g., WHO and European Medicines Agency)].

The questionnaire was pretested. The pretesting involved 20 parents, who were not part of the final sample, to assess the clarity of the questions. No changes were made to the instrument following this assessment.

### 2.4. Sample Size

Based on the prevalence of VH and refusal [27,28,30,31,32], a minimum sample size of 352 subjects was calculated using the Raosoft sample size calculator [33] providing a confidence level of 95% with a margin of error of 5%. Based on the response rate from previous studies in the same population [15,27,34,35,36,37], the total sample size was inflated to at least 1150 individuals.

### 2.5. Statistical Analysis

The mean and standard deviation (SD) were used to describe continuous data when normally distributed. The median and interquartile range (IQR) were used in case of deviations from normality. Frequencies were used to summarize categorical data. The skewness of the variables was estimated by Shapiro–Wilk tests. A bivariate analysis to evaluate the relationship between each independent characteristic and the various outcomes of interest was conducted. For continuous variables, if samples were normally distributed, Student’s t-test (two groups) and one-way ANOVA (>2 groups) were used, whereas the Wilcoxon–Mann–Whitney (two groups) and the Kruskal–Wallis (>2 groups) tests were used if normality was violated. For categorical variables, Pearson’s chi-square test was used.

Multinomial and multiple logistic regression models were developed according to the Hosmer and Lemeshow strategy [38], and independent variables with a *p*-value of 0.25 or less in the bivariate analysis were included in the models. Scientifically relevant variables were also included in the models, regardless of the results of the bivariate analysis, and the rationale for this approach was to provide as complete control of confounding as possible within the given dataset. A multinomial logistic regression analysis was performed to investigate CPVH categorized into three levels on the basis of the score obtained through the PACV-5 scale [non/low-CPVH (PACV-5 score = 0–4) (baseline group), moderate CPVH (PACV-5 score = 5–6) and high CPVH (PACV-5 score = 7–10)] (Model 1). A multiple logistic regression analysis was employed to investigate the dichotomous outcome of the parent having refused the COVID-19 vaccination for their child (no = 0; yes = 1) (Model 2). The following independent parent’s variables were included in both models: age in years (continuous), sex (male = 0; female = 1), marital status (unmarried/separated/divorced/widowed = 0; married/cohabiting with a partner = 1), education level (high secondary school or lower = 0; academic degree or higher = 1), working in the healthcare sector (no = 0; yes = 1), parental COVID-19 vaccination status (no = 0; yes = 1), knowledge score about COVID-19 and its vaccination in children (continuous), concern that their child can get sick with COVID-19 (no = 0; yes = 1), belief that their child can transmit COVID-19 to family and friends (no = 0; yes = 1) and that non-pharmacological interventions (e.g., facemasks) are enough to protect the child from COVID-19 (no = 0; yes = 1), sources of information on COVID-19 vaccination used and trusted [none = 0; informal sources = 1; both formal and informal sources = 2, formal sources = 3], and need for further information about COVID-19 vaccination (no = 0; yes = 1). In Model 2, the variable CPVH [non/low/moderate (PACV-5 score = 0–6) = 0; high (PACV-5 score = 7–10) = 1] was also included. The results of the models are presented as coefficients (Coeff) or Odds Ratios (ORs) with 95% confidence intervals (CIs). For all analyses, two-tailed tests were used, and a *p*-value equal to or less than 0.05 was considered statistically significant.

Statistical analysis was developed using Stata Statistical Software, Version 19.0 [39].

## 3. Results

Overall, 511 parents of children aged 5–11 years provided a questionnaire reply. Five paper questionnaires with more than 20% missing data were excluded from the study; hence, the final sample consisted of 506 parents. Among parents who potentially had access to the online version of the survey (nearly 677), 253 completed the questionnaire with an estimated response rate of 37.4%. Among parents who had access to the paper version of the survey (389), 253 completed it (response rate of 65%). The majority (69.2%) of the sample were female, and the median age of the sample was 38 years (IQR 34–42). More than half (54.7%) held an academic degree, and almost three-quarters were married (73.8%) and employed (71.3%); among those, almost a quarter (21.7%) were employed in the healthcare sector. The mean age of the children included in the sample was 7.9 years (±2 SD), and the number of male and female children was roughly the same.

### 3.1. Parents’ Knowledge About COVID-19 in Children

As displayed in Table 1, 43.3% of the sample were aware that COVID-19 could have severe consequences for children and 37.2% that it increases the risk of hospitalization among unvaccinated children and/or those with underlying conditions. Only 27.7% of parents knew that it is estimated that 10% of children who get COVID-19 develop a syndrome characterized by the persistence of clinical signs and symptoms of the infection (so-called long COVID); 21.5% recognized that the Omicron variant of COVID-19 could pose a greater threat to children. Only one-third correctly answered that the COVID-19 vaccine is effective (34.2%) and safe (32.2%) in children aged 5 to 11 years. The median knowledge score was 1 (IQR 0–4), and only 12.7% correctly answered all six statements.

### 3.2. Concerns and Beliefs About COVID-19 and Immunization

About two-fifths (42.9%) of the parents disclosed high concern about the chance their child can get sick with COVID-19, and three-fifths (61.5%) believed that their child can transmit the infection to family and friends, as shown in Table 1. Among the participants, 40.9% believed that non-pharmacological interventions (e.g., facemasks) are enough to protect the child from COVID-19. More than half (55.7%) believed that an excessive amount of information about COVID-19 did not hinder the adherence to vaccination.

### 3.3. CPVH Based on the PACV-5 Score

The distribution of the responses for each item of the PACV-5 is presented in Table 2. More than half of the parents agreed that children get more shots than are good for them (64.2%) and that it is better to get fewer vaccines at the same time (69.9%). About half (49.2%) of the sample thought that it is better for children to develop immunity by getting sick than to get a shot, whereas just 32.8% trusted the information they received about COVID-19 vaccination. More than half (62.6%) considered themselves to be hesitant regarding COVID-19 vaccination. Overall, the median score of the PACV-5 was 8 (IQR 5–10), and high and moderate CPVH were found in 60.1% and 16.8% of the sample, respectively.

### 3.4. Bivariate and Multinomial Logistic Regression Analyses on CPVH

The results from the bivariate analysis presented in Table 1 show that high CPVH was significantly associated with virtually all of the explored variables. The multinomial logistic regression analysis (Model 1, Table 3) showed that parents with higher knowledge score were associated with lower odds of being classified as parents moderately (Coeff = −0.42; 95% CI: −0.60–0.23) or highly hesitant toward COVID-19 vaccination (Coeff = −0.46; 95% CI: −0.63–0.28). Having an academic degree or higher significantly reduced the likelihood of moderate CPVH (Coeff = −0.84; 95% CI: −1.58–0.11). In addition, older parents were less likely to have high CPVH (Coeff = −0.07; 95% CI: −0.11–0.02). The type of information source used was a strong predictor of CPVH. Compared with parents who did not receive information about COVID-19 vaccination, those who relied on formal sources had a substantially lower likelihood of moderate (Coeff = −1.44; 95% CI: −2.28–0.59) and high (Coeff = −1.74; 95% CI: −2.88–0.90) CPVH. Similarly, parents who used both formal and informal sources also showed reduced likelihood of moderate (Coeff = −1.93; 95% CI: −2.90–0.96) and high (Coeff = −1.45; 95% CI: −2.33–0.58) CPVH. In contrast, parents who received and who had trusted in information from informal sources were more likely to be classified as parents highly hesitant toward COVID-19 vaccination (Coeff = 1.48; 95% CI: 0.40–2.55).

### 3.5. COVID-19 Vaccination Status, Refusal, and Intention

The majority (78.5%) of the parents reported having received the COVID-19 vaccination. More than half of the parents (58.3%) stated that their child was vaccinated against COVID-19; the most cited reasons were to facilitate their child to attend school (59%), to avoid severe consequences caused by COVID-19 (42%), belief that the vaccine is an effective method to prevent COVID-19 (34.2%), having received a pediatrician’s recommendation to immunize the child (33.6%), and trust in vaccinations (20.5%). Among parents of unvaccinated children, only 7.6% intended to vaccinate their child, and the most reported reason was trust in vaccinations (37.5%), followed by avoiding severe consequences caused by COVID-19 (25%) and belief that the vaccine is an effective method to prevent COVID-19 (25%) and to facilitate their child to attend school (25%).

Almost two-fifths (38.5%) had no intention to vaccinate their child against COVID-19. The main reasons behind parental refusal were having no trust in vaccinations (48.2%) and considering developing immunity by getting sick better than getting a shot (45.1%). Additional reasons were having not received a pediatrician’s recommendation (31.3%), belief that vaccine is unnecessary since their child is unlikely to get very sick from COVID-19 (21.5%), needing further information on the COVID-19 vaccine (18%) and fear of the potential vaccination side effects (14.9%).

### 3.6. Bivariate and Multiple Logistic Regression Analysis on Parental COVID-19 Vaccination Refusal

The results from the bivariate analysis presented in Table 1 showed that COVID-19 vaccination refusal was significantly associated with virtually all of the explored variables. The logistic regression model (Model 2, Table 4) showed that high CPVH (OR: 3.10; 95% CI: 1.64–5.88) increased by 3.1-fold the odds of COVID-19 vaccination refusal, as expected. Similarly, needing further information on COVID-19 vaccination (OR: 2.11; 95% CI: 1.13–3.94) was a significant predictor of COVID-19 vaccination refusal. On the other hand, parents who were vaccinated against COVID-19 (OR: 0.13; 95% CI: 0.07–0.26) and those who received and trusted information on COVID-19 vaccination from formal sources (OR: 0.31; 95% CI: 0.13–0.74) and from both formal and informal ones (OR: 0.94; 95% CI: 0.44–1.99) were less likely to refuse the COVID-19 vaccine compared with those who did not receive information at all. Furthermore, for each one-point increase in the knowledge score (OR: 0.55; 95% CI: 0.46–0.68), the odds of parental COVID-19 vaccine refusal decreased by a factor of 0.55.

### 3.7. Source of Information

About two-fifths (40.9%) of the parents declared to have received information on COVID-19 vaccination only from informal sources and trusted these sources, 21.3% from formal sources and 16.8% from both formal and informal ones (Table 1). One-third (33.8%) of the sample needed additional information about COVID-19 vaccination.

## 4. Discussion

This study provides valuable insights into CPVH and refusal among parents of children aged 5–11 in the southern part of Italy. The study highlighted a notably high proportion of parents were classified as highly hesitant, despite most being vaccinated themselves. Among predictors of CPVH and refusal, lower levels of knowledge about COVID-19 and immunization and using informal information sources were identified. CPVH and refusal were also deeply interconnected with individual beliefs, perception of risks and benefits of vaccination, and concerns about vaccine safety.

One of the key findings from our study is the significant proportion of parents (60.1%) who were classified as highly hesitant. Similarly, surveys among parents performed early in the pandemic or shortly after pediatric COVID-19 vaccines became available showed high levels of hesitancy [40,41,42,43]. The high level of hesitancy reflected the uncertainty and safety concerns commonly observed during the rollout of new vaccines. A meta-analysis of Italian studies conducted one year before the present data collection reported slightly lower CPVH levels [18]. International estimates also vary widely: CPVH prevalence reaches 43.3% in the United States [44], approximately 33% in Malaysia [45], and 20.4% in a European cohort of parents of children and adolescents under 18 years. More specifically, the rate was 25.2% in Poland, followed by 22.6% in Italy, 22% in Portugal, and 11.1% in Spain. These cross-country differences could be explained by the influence of strong anti-vaccination movements (e.g., Poland) [46] or by high levels of skepticism regarding the importance of vaccines and concerns about their effectiveness (e.g., Italy) [46].

However, subsequent surveys carried out when pediatric vaccination for COVID-19 had yet been in place also showed high rate of hesitancy towards the COVID-19 vaccine for children [47]. The expectation that some of the earlier concerns expressed by parents regarding the vaccine’s safety, novelty, and efficacy would have been alleviated after more years of pediatric COVID-19 vaccination availability was disregarded, and routine childhood vaccinations seem to receive remarkable acceptance [47].

It is interesting to note that nearly half of the parents remained hesitant to vaccinate their children, despite the high COVID-19 vaccination rates (76.5%). However, having not received the COVID-19 vaccination was one of the predictors of CPVH in the bivariate analysis. The latter figure seems to suggest that not all vaccinated parents are motivated to vaccinate their children, although available evidence suggests that parents are more inclined to vaccinate their children if they have been vaccinated themselves [48,49,50,51,52]. Parents take more into consideration than ever before when making vaccine-related decisions for their children. This may be because COVID-19 vaccines have brought many aspects of vaccination development, testing, and novel technologies into the public eye. Specifically, for the COVID-19 vaccine, concern for long term side-effects in children and a desire for more long-term research represent an area of hesitation. Additionally, parents could have safety concerns for children because they are still in their developmental stages of life.

A lower level of knowledge about COVID-19 and immunization in children is a significant predictor of CPVH at the multinomial regression analysis. The fact that many parents in our sample were unaware of the risks that COVID-19 poses to unvaccinated children (i.e., severe side effects, increased hospitalization rates, and long COVID), coupled with misconceptions about the vaccine’s effectiveness and safety, highlight significant gaps to be filled. It could be argued that these knowledge gaps are the result of the COVID-19 infodemic emergency, a phenomenon that increases with the rapid rollout of new vaccines and reflects a missed opportunity in public health communication. It is also noteworthy that one demographic characteristic was associated with CPVH. Indeed, a younger age of parents seems to predict their hesitancy to vaccinate their children against COVID-19. Hence, future communication strategies should prioritize raising awareness, especially of young parents, about the risks of COVID-19, as well as other vaccine-preventable diseases. Indeed, evidence shows that individuals often give more weight to risks of side effects of the vaccine than severity of or susceptibility to a disease when making decisions [7,53]. Focusing on parents, spending more time with young parents, and supporting them through the decision-making process could effectively reduce doubts and misinformation, preventing the shift to refusal.

Nearly two-fifths of parents reported that their children had not received vaccination against COVID-19, and, among those, only 7.6% disclosed their willingness to get their child vaccinated. Similarly, with CPVH, the belief that children should acquire immunity by contracting the disease rather than through vaccination alongside a general distrust in vaccinations and concerns regarding vaccine safety were among the more frequent reasons for refusal. A growing body of evidence has shown that some parents believe that vaccines are “toxins” and natural exposure provides more long-lasting immunity [54]. In addition, vaccine refusal has been linked to moral convictions and philosophical beliefs, such as a preference for natural over artificial medicines. It may be important, for specific populations, to establish community and religious influences to profitably deliver vaccination information and tailor specific interventions [55].

Interestingly, a lack of recommendation by HCPs is one of the reasons most reported by parents for not getting their child vaccinated; likewise, following a HCP’s recommendation was one of the more frequently cited reasons for parents having their child vaccinated against COVID-19. Importantly, the present study highlights the crucial role of information sources in shaping vaccine refusal. Indeed, parents who relied on HCPs’ vaccine-related information were significantly less likely to refuse COVID-19 vaccination for their children, whereas those who relied on informal sources, such as social media or family and friends, demonstrated higher levels of vaccine refusal. Research consistently showed the value of tailored communication, especially when it is delivered by trusted HCPs within a strong patient–HCP relationship, in significantly enhancing vaccine acceptance [56]. The study results highlighted the importance of equipping HCPs with the tools and resources necessary to communicate effectively about vaccine safety and efficacy, especially with hesitant parents.

Moreover, much attention has been paid to the role of social media in forming parental views around vaccinations and its use as a vehicle to spread myths and negative views on vaccinations. Reliance on social media platforms for vaccine-related information was associated with a higher likelihood of CPVH [57]. This trend coincided with the proliferation of misinformation and conspiracy theories that depicted COVID-19 vaccines as harmful during the pandemic. Similar patterns were observed for other types of vaccines, including polio; measles, mumps, and rubella; and diphtheria–tetanus–pertussis vaccines [57]. However, social media platforms can be a powerful tool for HCPs and policymakers to promote informed decision-making. Efforts focused on employing social media platforms to disseminate trustworthy information and combat hesitancy could help to foster a well-informed population and, ultimately, increasing vaccine uptake [58]. One example of the success in utilizing social media platforms is Ireland’s achievement in restoring public confidence in the human papillomavirus (HPV) vaccination, as demonstrated through YouTube and Facebook [59]. Similarly to COVID-19 vaccines, a desire for more long-term research represented an area of hesitation among parents when the HPV vaccine was released [52]. Moreover, the need for additional information about COVID-19 vaccines emerged as a predictor of refusal. A desire for more details about vaccine safety and efficacy underscores the urgent need to enhance the accessibility of official health communication.

### Limits

To appreciate the findings of this study, some limitations must be acknowledged. Firstly, the cross-sectional study design does not permit the establishment of a cause-and-effect relationship between the predictor variables and the dependent variables. Nevertheless, this study can describe general associations. Secondly, self-reporting of information may have heightened the likelihood of bias, such as social desirability bias, as participants may have consciously chosen responses that favor vaccination, which might not accurately reflect their true behavior. However, one of the means for limiting this bias includes adherence to procedures that maximize anonymity and confidentiality, as was performed in the survey. Third, concerns about the generalizability of our results may arise. The external validity of the study, especially the temporal validity, may be questioned, and the generalization of the findings across time could be limited. However, to address and prevent the growing phenomenon of VH, it is imperative to identify its nature and extent in the local context. Moreover, it could be argued that minimal modifications might have occurred since, from the time of data collection to today, minimal interventions have been put in place to increase vaccination uptake in this population group. Moreover, the study findings can support future pandemic scenarios by providing insight into how to appropriately respond when a new vaccine is introduced. Fourth, the estimates of study population characteristics were partially obtained through an online survey available on the school’s website home page since methodological issues, such as under-coverage, may arise. We have no way of knowing exactly the number of parents who logged into the school website and saw the link to the online survey, but we can estimate that, among the total number of eligible students’ parents (903), nearly 25% did not have access to the school’s website home page and/or to the electronic class register during the data collection period, meaning a response rate close to 40%. However, to reduce under-coverage issues, we even applied paper surveys to include those individuals who lack Internet access or are less tech-savvy. Fifth, female parents predominate in answering the questions, and different opinions or patterns of willingness to vaccinate their children against COVID-19 may exist among parents. However, it should be pointed out that mothers usually undertake most childcare duties and make decisions and arrangements regarding immunization [16,60]. Therefore, it is unlikely that the over-representation of female parents may distort the estimate of hesitancy toward and refusal of vaccination against COVID-19 for their 5–11-year-old children.

## 5. Conclusions

This study provides valuable insights into CPVH and refusal among parents of children aged 5–11 in Italy, identifying factors contributing to parental negative attitudes and intentions toward COVID-19 vaccination. The paper argued that CPVH is fueled by knowledge gaps and a lack of HCP guidance or recommendation, and these figures open a window of opportunity for intervention and confirm the importance of considering those factors while planning public health interventions in case of similar future events. The findings highlight the need for establishing and investing in social platforms to promote vaccine awareness and dispel misinformation among parents while creating tools capable of monitoring the sentiment on the topic. In order to be prepared for future similar events, public health systems need to constantly boost vaccine confidence among parents by increasing HCP engagement in conversations about vaccines and vaccination. In the context of a public health emergency, such as a pandemic, it is necessary to strengthen the trust of the population through the implementation of health communication and public education strategies. Therefore, public health systems should invest in training all the HCPs in learning adaptive communication strategies that assimilate from parents’ feedback, adjust messaging strategies, or respond to their evolving concerns in real time.

## Figures and Tables

**Table 1:** Distribution of potential determinants of parental vaccine hesitancy and vaccine refusal regarding COVID-19 vaccination.

Potential Determinants	Responses	Overall Sample (*n* = 506)		CPVH as PACV-5 Score (*n* = 506)							Vaccine-Refusing Parents (*n* = 195)		
				Non-Hesitant/ Low-Hesitancy (0–4)		Moderately Hesitant (5–6)		Highly Hesitant (7–10)		*p*-Value	Refusal		*p*-Value
		*n*	(%)	*n*	(%)	*n*	(%)	*n*	(%)		*n*	(%)	
*Sociodemographic characteristics*													
Sex	Female	350	69.2	88	75.2	62	72.9	200	65.8	0.122	126	64.6	0.079
Age	Median (IQR)	38	(34–42)	40	(37–44)	38	(33–43)	37	(34–41)	0.0003	38	(34–42)	0.492
Marital Status	Married/cohabiting with a partner	373	73.7	97	82.9	67	78.82	209	68.75	0.006	141	72.3	0.569
Educational level	Academic degree or higher	277	54.7	77	65.8	40	47.1	160	52.6	0.015	105	53.8	0.748
Working in the healthcare sector	Yes	110	21.7	35	29.9	19	22.4	56	18.4	0.037	30	15.4	0.006
Parental COVID-19 vaccination	Yes	397	78.5	117	100	79	93	201	66.1	<0.001	101	51.8	<0.001
*Knowledge about COVID-19 and immunization in children*													
COVID-19 increases hospitalization among unvaccinated children and/or with underlying conditions	True	188	37.2	77	65.8	39	45.9	72	23.7	<0.001	22	11.3	<0.001
COVID-19 could have serious consequences for children	True	219	43.3	84	71.8	44	51.8	91	29.9	<0.001	28	14.4	<0.001
COVID-19 vaccine is safe in children aged 5 to 11	True	163	32.2	86	73.5	29	34.1	45	15.8	<0.001	9	4.6	<0.001
COVID-19 vaccine is effective in children aged 5 to 11	True	173	34.2	84	71.8	35	41.2	54	17.8	<0.001	13	6.7	<0.001
Omicron variant of COVID-19 could pose a greater threat to children	True	109	21.5	47	40.2	20	23.5	42	13.8	<0.001	9	4.6	<0.001
It is estimated that 10% of children who get COVID-19 develop long COVID	True	140	27.7	60	51.3	28	32.9	52	17.1	<0.001	15	7.7	<0.001
Knowledge score	Median (IQR)	1	(0–4)	4	(2–6)	2	(1–4)	0	(0–2)	0.0001	0	(0–0)	<0.001
*Concerns and beliefs about COVID-19 and immunization*													
Concern that their child can get sick with COVID-19	Low (0–4)	179	35.4	17	14.5	12	14.1	150	49.3	<0.001	118	60.5	<0.001
	Moderate (5–6)	110	21.7	22	18.8	24	28.2	64	21.1		36	18.5	
	High (7–10)	217	42.9	78	66.7	49	57.7	90	29.6		41	21	
Belief that the child can transmit COVID-19 to family and friends	Agree ^a^	311	61.5	97	82.9	64	75.3	150	49.3	<0.001	87	44.6	<0.001
	Not sure	56	11	10	8.6	9	10.6	37	12.2		23	11.8	
	Disagree ^b^	139	27.5	10	8.5	12	14.1	117	27.5		85	43.6	
Belief that non-pharmacological interventions (e.g., facemasks) are enough to protect the child from COVID-19	Agree ^a^	207	40.9	76	65	50	58.8	81	26.6	<0.001	45	23.1	<0.001
	Not sure	122	24.1	28	23.9	25	29.4	69	22.7		36	18.4	
	Disagree ^b^	177	35	13	11.1	10	11.8	154	50.7		114	58.5	
Belief that excessive information may lead to decreased adherence to COVID-19 vaccination	Yes	224	44.3	55	47	35	41.2	134	44.1	0.708	88	45.1	0.758
*Sources of information and trust*													
Need for further information about the COVID-19 vaccination	Yes	171	33.8	51	43.6	36	42.4	84	27.6	0.002	54	27.7	0.026
Sources of information on COVID-19 vaccination used and trusted	Formal sources ^c^	108	21.3	54	46.2	26	30.6	28	9.2	<0.001	11	5.6	<0.001
	Informal sources ^d^	207	40.9	7	6	17	20	183	60.2		123	63.1	
	Both formal and informal sources ^c,d^	85	16.8	41	35	12	14.1	32	10.5		13	6.7	
	None	106	21	15	12.8	30	35.3	61	20.1		48	24.6	

^a^ Agree shows combined responses of strongly agree and agree. ^b^ Disagree shows combined responses of strongly disagree and disagree. ^c^ Formal sources include pediatricians, other HCPs, and national (e.g., Italian Ministry of Health, National Health Institute) and international health organizations (e.g., WHO, EMA). ^d^ Informal sources include mass media, family and friends, the Internet, and social media. CPVH = COVID-19 parental vaccine hesitancy, PACV-5 = Parent Attitudes about Childhood Vaccines short scale.

**Table 2:** Responses to individual PACV-5 items about COVID-19 vaccine.

Item	Agree ^a^		Unsure		Disagree ^b^	
	*n*	%	*n*	%	*n*	%
Children get more shots than are good for them	325	64.2	97	19.2	84	16.6
It is better for my child to develop immunity by getting sick than to get a shot	249	49.2	122	24.1	135	26.7
It is better for children to get fewer vaccines at the same time	352	69.6	91	18	63	12.4
I trust the information I receive about COVID-19 vaccination	166	32.8	126	24.9	214	42.3
	**Hesitant ^c^**		**Unsure**		**Not hesitant ^d^**	
	***n***	**%**	***n***	**%**	***n***	**%**
Overall, how hesitant about COVID-19 vaccination would you consider yourself to be?	317	62.6	84	16.6	105	20.8

^a^ Agree shows combined responses of strongly agree and agree. ^b^ Disagree shows combined responses of strongly disagree and disagree. ^c^ Hesitant shows combined responses of very and somewhat hesitant. ^d^ Not hesitant shows combined responses of not at all and not too hesitant. PACV-5 = Parent Attitudes about Childhood Vaccines short scale.

**Table 3:** Results of the multinomial logistic regression model for potential predictors of CPVH.

**Outcome: CPVH (Non/Low CPVH as Baseline Group)** *Log Likelihood = −326.59399; Prob > chi2 = 0.0000; Obs = 506*						
**Variables**	**Moderate CPVH**			**High CPVH**		
	**Coeff**	**95% CI**	***p*-Value**	**Coeff**	**95% CI**	***p*-Value**
**Sex**						
Male *	1.00			1.00		
Female	−0.49	−1.26–0.28	0.214	0.46	−1.18–0.25	0.206
**Age in years, continuous**	−0.04	0.09–0.01	0.092	−0.07	−0.11–0.02	0.005
**Marital status**						
Single/divorced/widowed *	1.00			1.00		
Married/cohabiting with a partner	−0.22	−1.02–0.58	0.588	−0.63	−1.35–0.89	0.086
**Educational level**						
High secondary school or lower *	1.00			1.00		
Academic degree or higher	−0.84	−1.58–0.11	0.024	−0.34	−1.03–0.35	0.330
**Working in the healthcare sector**						
No *	1.00			1.00		
Yes	0.38	0.46–1.22	0.372	0.36	−0.42—1.14	0.360
**Parental COVID-19 vaccination**						
No *	1.00			1.00		
Yes	−13.92	−1265–1237	0.983	−15.43	−1266–1236	0.981
**Knowledge score about COVID-19 and its vaccination in children, continuous**	−0.42	0.60–0.23	<0.001	−0.46	−0.63–0.28	<0.001
**Concern that their child can get sick with COVID-19**						
No *	1.00			1.00		
Yes	0.17	0.53–0.86	0.637	−0.02	−0.67–0.63	0.943
**Belief that their child can transmit COVID-19 to family and friends**						
No *	1.00			1.00		1.00
Yes	0.31	0.54–1.16	0.471	−0.01−	−0.78–0.76	0.979
**Belief that non-pharmacological interventions (e.g., facemasks) are enough to protect the child from COVID-19**						
No *	1.00			1.00		
Yes	0.55	0.16–1.27	0.130	−0.17−	−0.83–0.48	0.603
**Need for further information about the COVID-19 vaccination**						
No *	1.00			1.00		1.00
Yes	−0.16	0.81–0.49	0.630	0.01	−0.60–0.61	0.991
**Sources of information on COVID-19 vaccination used and trusted**						
None *	1.00			1.00		
Informal sources	0.15	−1.03–1.34	0.798	1.48	0.40–2.55	0.007
Both formal and informal sources	−1.93	−2.90–0.96	<0.001	−1.45	−2.31–0.58	<0.001
Formal sources	−1.44	−2.28–0.59	<0.001	−1.74	−2.58–0.91	<0.001

CPVH = COVID-19 parental vaccine hesitancy; Log likelihood = log-likelihood function; Obs = observations; Prob = probability; chi2 = chi-square, Coeff = Coefficient; 95% CI = 95% confidence interval. * Reference category.

**Table 4:** Results of the logistic regression model for potential predictors of parents refusing the COVID-19 vaccination for their child.

**Variables**	**OR**	**95% CI**	***p*** **-Value**
**Outcome: Parent refusing the COVID-19 vaccination for their child** *Log likelihood = −195.76564; Prob > chi2 = 283.10; Obs = 506*			
**CPVH**			
Non/low/moderate *	1.00		
High	3.10	1.64–5.88	<0.001
**Parental COVID-19 vaccination**			
No *			
Yes	0.13	0.07–0.26	<0.001
**Knowledge score about COVID-19 and its vaccination in children, continuous**	0.55	0.46–0.68	<0.001
**Sources of information on COVID-19 vaccination used and trusted**			
None *	1.00		
Informal sources	0.94	0.44–1.99	0.864
Both formal and informal sources	0.33	0.13–0.82	0.017
Formal sources	0.31	0.13–0.74	0.009
**Need for further information about the COVID-19 vaccination**			
No *	1.00		
Yes	2.11	1.13–3.94	0.019
**Marital status**			
Single/divorced/widowed *	1.00		
Married/cohabiting with a partner	1.53	0.85–2.77	0.156
**Educational level**			
High secondary school or lower *	1.00		
Academic degree or higher	1.47	0.84–2.59	0.178
**Working in the healthcare sector**			
No *	1.00		
Yes	1.61	0.78–3.34	0.197
**Belief that non-pharmacological interventions (e.g., facemasks) are enough to protect the child from COVID-19**			
No *	1.00		
Yes	1.39	0.71–2.75	0.340
**Concern that their child can get sick with COVID-19**			
No *	1.00		
Yes	0.80	0.43–1.52	0.504
**Concern that their child can transmit COVID-19 to family and friends**			
No *	1.00		
Yes	0.86	0.45–1.66	0.658
**Sex**			
Male *	1.00		
Female	0.95	0.53–1.68	0.850
**Age in years, continuous**	1.00	0.96–1.04	0.863

CPVH = COVID-19 parental vaccine hesitancy; Log likelihood = log-likelihood function; Obs = observations; Prob = probability; chi2 = chi-square, OR = Odds Ratio; 95% CI = 95% confidence interval. * Reference category.

## Data Availability

The data presented in this study are openly available in the Mendeley Data repository (doi:10.17632/67xmhjhk9t.4).

## Abbreviations

The following abbreviations are used in this manuscript:

CIs	Confidence intervals
Coeff	Coefficient
CPVH	COVID-19 parental vaccine hesitancy
HCPs	Healthcare providers
MIS-C	Multisystemic inflammatory syndrome in children
ORs	Odds Ratios
PACV-5	Parent Attitudes about Childhood Vaccines short scale
PVH	Parental vaccine hesitancy
SD	Standard deviation
STROBE	Strengthening the Reporting of Observational Studies in Epidemiology
VH	Vaccine hesitancy
WHO	World Health Organization
